# Supplementary material for: Thyroid Hormone Replacement Therapy Is Associated with Longer Overall Survival in Patients with Resectable Gastroesophageal Cancer: A Retrospective Single-Center Analysis
Source: Cancers (Basel). 2021 Oct 9;13(20):5050. doi: 10.3390/cancers13205050 (PMC8534173; doi:10.3390/cancers13205050)
Supplement: Supplementary file 1 [file cancers-13-05050-s001.zip › cancers-1408912-supplementary.pdf]

Supplementary Figure S1: Tumor location and histological subtype.
GEJ=gastroesophageal junction

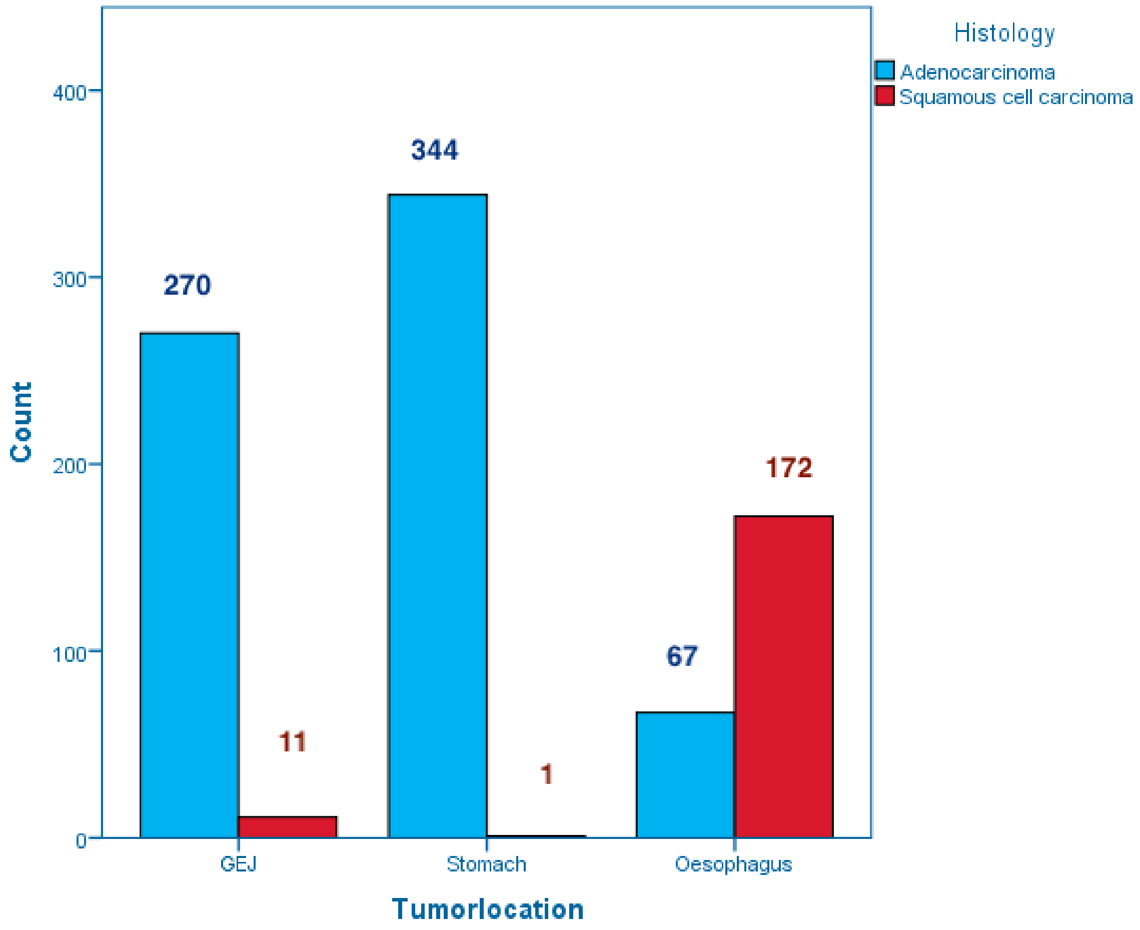

Supplementary Figure S2: Kaplan Meier curves of demographic and cancer specific characteristics

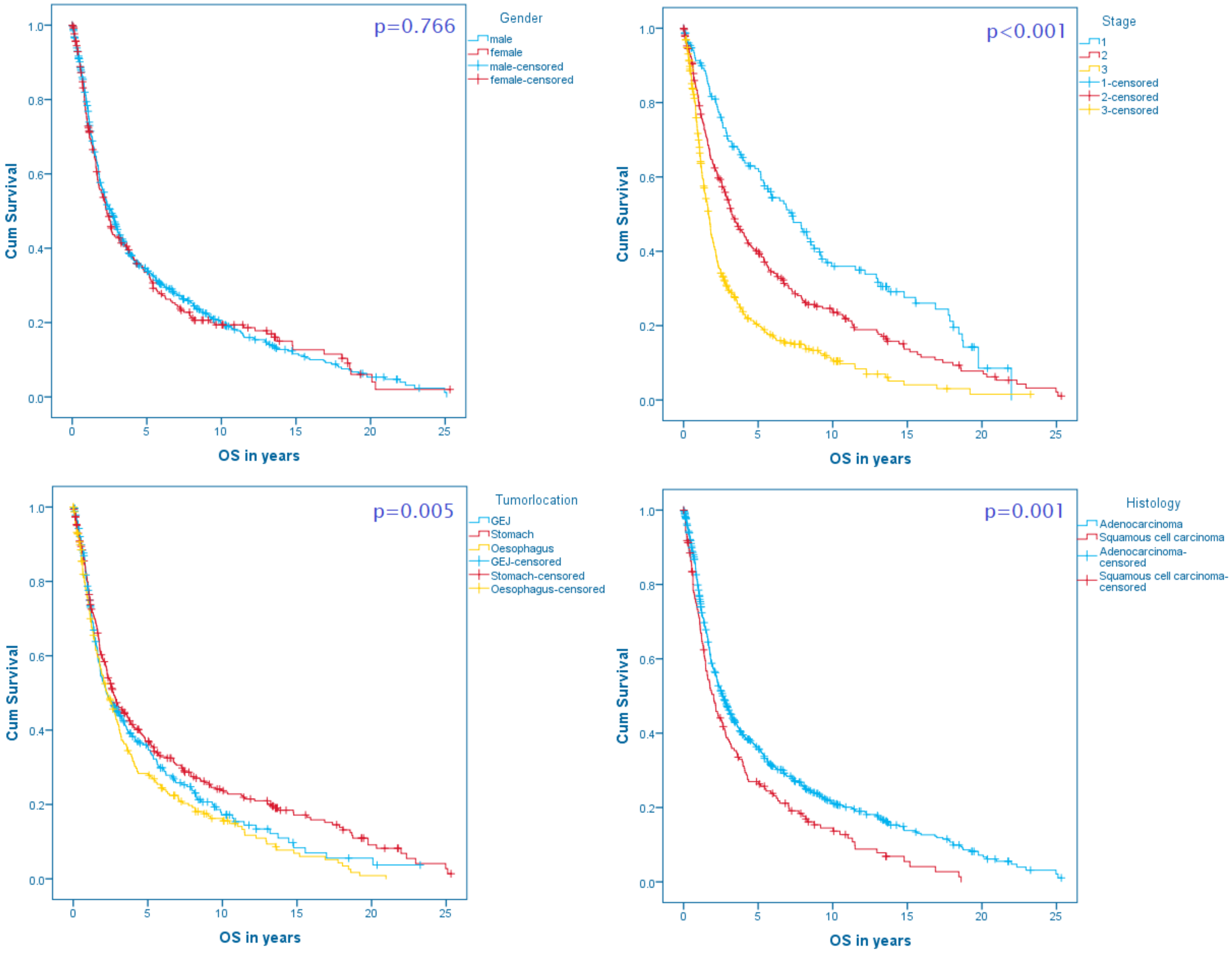

Supplementary Figure S3: Boxplots of thyroid hormones in the overall cohort

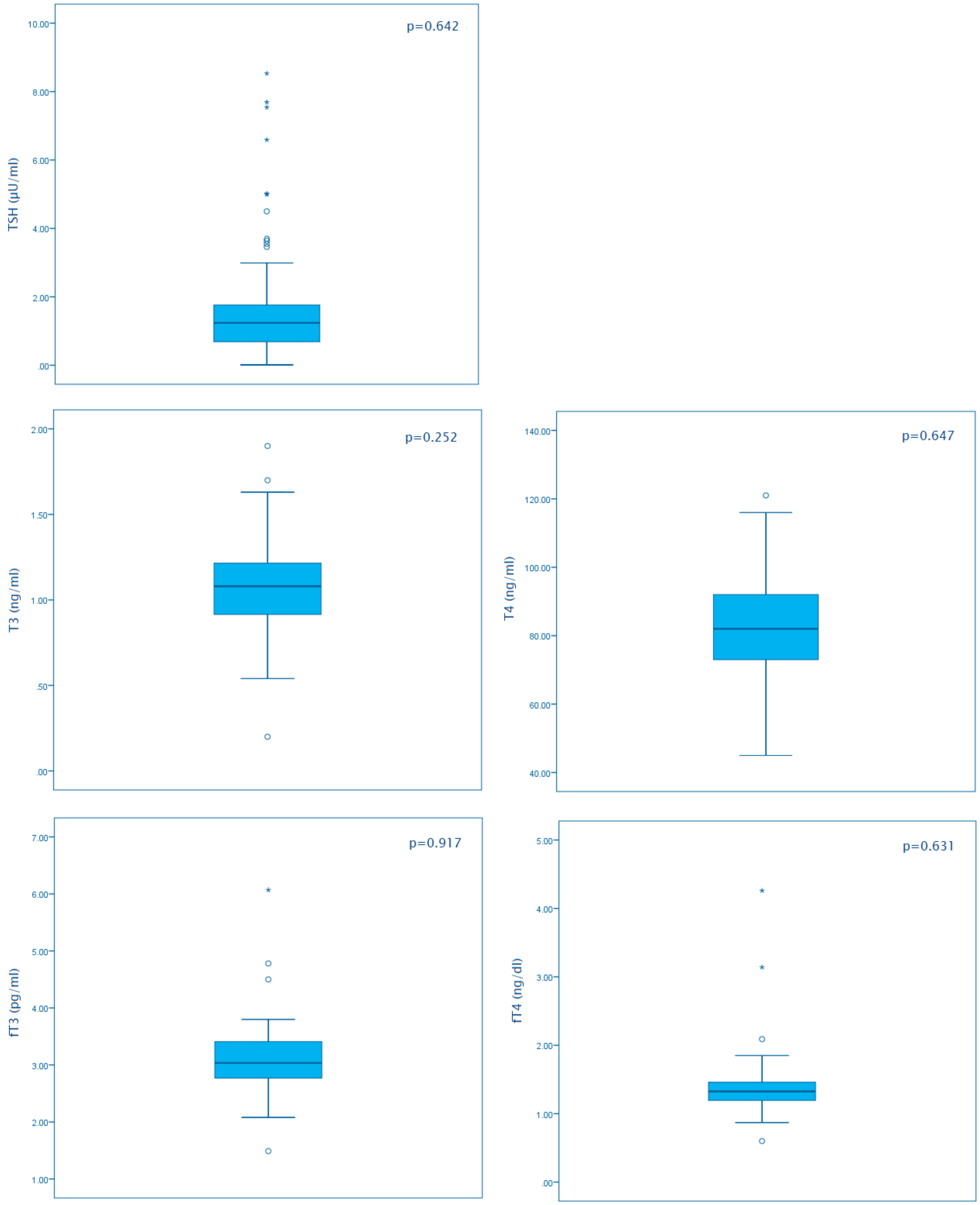

Supplementary Figure S4: Clinical relevance of thyroid disorders

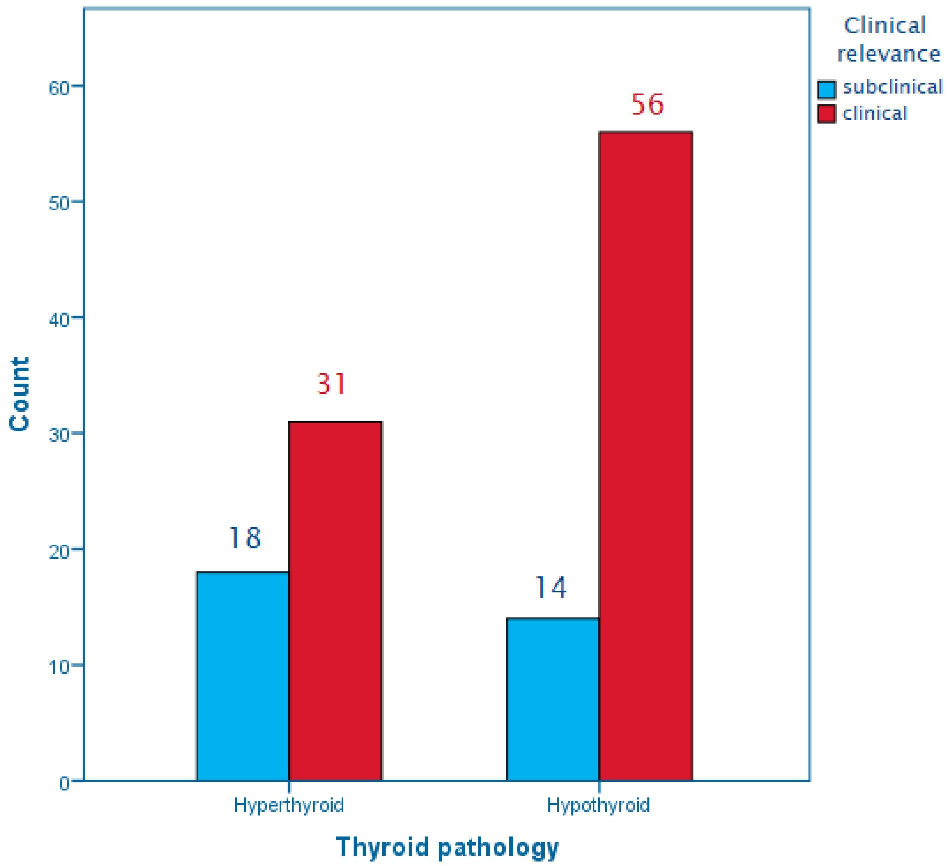

Supplementary Figure S5: Thyroid disorders regarding tumor stages.

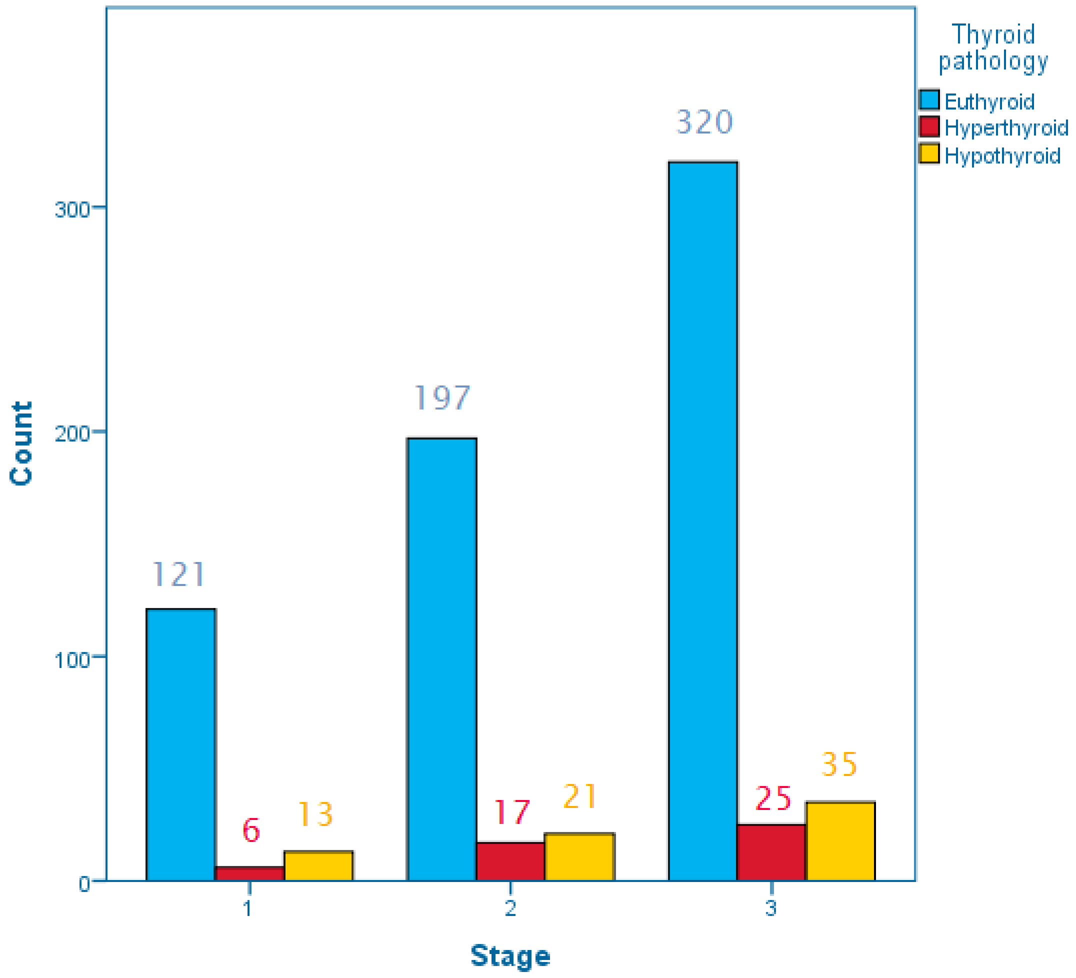

Supplementary Table S1: Missing endocrinological values

Characteristic	Data available	Data missing
**TSH (**μU/ml)		
n (%)	209 (24.2%)	656 (75.9%)
**T3 (**ng/ml)		
n (%)	60 (6.9%)	805 (93.1%)
**T4 (**ng/ml)		
n (%)	89 (10.3%)	776 (89.7%)
**fT3** (pg/ml)		
n (%)	38 (4.4%)	827 (95.6%)
**fT4 (**ng/dl)		
n (%)	80 (9.2%)	785 (90.8%)
**Thyroid pathology**		
n (%)	767 (88.7%)	98 (11.3%)
